# Mersey deanery ophthalmology trainees’ views of the objective assessment of surgical and technical skills (OSATS) workplace-based assessment tool

**DOI:** 10.1007/s40037-013-0041-8

**Published:** 2013-02-01

**Authors:** Myrto Tsagkataki, Anshoo Choudhary

**Affiliations:** 1 St Paul’s Eye Unit, Royal Liverpool and Broadgreen University Hospitals NHS Trust, Mersey Deanery, Prescot Street, Liverpool, L7 8XP UK; 2 St Paul’s Eye Unit, Royal Liverpool and Broadgreen University Hospitals NHS Trust, Liverpool, L7 8XP UK

**Keywords:** Workplace based assessments, Objective assessment of surgical and technical skills, Ophthalmology training, Cataract surgery

## Abstract

Objective assessment of surgical and technical skills (OSATS) workplace-based assessment tool is now mandatory during ophthalmology speciality training in the United Kingdom. The opinions of those undergoing this assessment have not been formally sought. This study evaluated the views of ophthalmology trainees on OSATS assessment as applied to cataract surgery. A questionnaire was circulated to 34 ophthalmology speciality trainees of the Mersey deanery. A total of 28 responses were received. The most positive aspects of the process identified were feedback, learning and opportunity for reflective practice. The most negative aspects were time constraints, assessor’s availability and case selection. Of the trainees, 93 % mentioned that no previous agreed action was taken into consideration when filling in subsequent forms and their performance was not discussed in their annual summative assessment. This study highlights important aspects of trainees’ perceptions of OSATS. Trainees appreciate the formative aspects of OSATS assessment. Some problems came to light, which can be resolved by specification of standards, training of assessors, and commitment from both trainers and trainees. Changes are needed to allow demonstration of surgical progression with time. The issues identified here will be relevant to other specialities as well. A larger survey would be beneficial.

## Introduction

The quality of ophthalmic training is increasingly challenged by the convergence of a number of recent issues including public expectations, demand for safety and NHS service pressures leading to reduced overall training opportunities [1, 2]. The challenge for current ophthalmologists is to increase the quality of training with reduced training opportunities and time.

Until recently, surgical skills in the UK were not formally assessed. Modernising Medical Careers has led to modifications of the assessments for trainees leading up to the award of a certificate of completion of training. A trainee’s progression is assessed by documented acquisition of competencies through workplace-based assessments (WpBAs) throughout the training period and formally reviewed at the annual review of competence progression (ARCP) meeting, in common with other specialities [1].

One of the first formal WpBA to assess surgical skills in Ophthalmology was the Objective Assessment of Surgical and Technical Skills (OSATS) tool. It was introduced by the Royal College of Ophthalmologists in 2007 [3]. Cataract surgery is the most commonly performed procedure and the key index surgery for ophthalmologists and elicits a distinct learning curve. We aimed to gather the views of a sample of UK ophthalmology trainees on the OSATS WpBA as applied to cataract surgery through a questionnaire survey for its acceptability and to identify potential problems. To our knowledge there have been no previous reports from ophthalmology trainees in this field.

## Methods

Data were collected through a questionnaire conducted by the authors. The questionnaire consisted of 14 questions with binary responses such as difficulty with format, case selection and time available to perform the assessment and receive feedback, stress during the assessment and adaptability of the form (Appendix). These questions were open in order to encourage longer free-text responses. Respondents were asked to give both positive and negative aspects of OSATS to reduce the possibility of introducing bias. They were also asked to suggest any improvements given that the aim was to identify any problems and acquire solutions to them. Finally there was a prompt for ‘any other comments’, in case respondents had felt constrained by the questionnaire design. Information on the year of training, number of OSATS assessments performed by each trainee and the level of the assessor was requested. Trainees were informed that the study was for research purposes only and would not count in any way towards formal assessment or appraisal. The questionnaire was first circulated in an electronic format to 34 ophthalmic speciality trainees (OSTs) of Mersey deanery in December 2010. A reminder, with the questionnaire attached, was emailed on two occasions prior to the deadline. After each reply the answers were saved anonymously in a file. 4 weeks later only 16 responses were received; therefore the questionnaire was also circulated in a paper-based form. The quotations in the sections that follow were chosen as best illustrating the general themes that emerged. In order to avoid identifying individuals, quotations were anonymized.

## Results

A total of 28 (83 %) responses were received. All the OSTs had experienced OSATS at least once. There was an equal split between junior and senior trainees. In order to identify differences with seniority, respondents were split into two groups: group A (OST 1–3) and group B (OST 4–7). A total of 38 % from group A and 100 % from group B had undergone OSATS nine or more times. The level of the assessors was mainly consultants (82 %). Other assessors included specialist registrars or senior trainees (14 %) and staff grades (4 %). The majority of the trainees (84 % in group A and 80 % in group B) felt OSATS is adaptable for every surgical procedure. Of the trainees, 93 % reported no difficulties using the current format and had encountered no problems with the process. More than half of the responders (54 %) found it difficult to have the assessment done, mostly due to time constraints (73 %) and assessor availability (20 %) and some had difficulties with case selection (7 %). The majority of the trainees (79 %) reported that there was time available to complete the procedure. In group A 84 % and in group B 33 % felt that there was sufficient time to receive feedback. Half of the trainees (70 % in group A and 53 % in group B) reported feeling stressed during the assessment, which affected their performance in a negative way. None of the trainees had had to repeat an assessment for not meeting expectations. Of the responders 93 % mentioned that no previous agreed action was taken into consideration when filling in subsequent forms and their performance in OSATS was not discussed in their annual summative assessment (ARCP).

Half of the responders felt that OSATS helped them to improve their skills and identified some learning points such as ‘enlarging capsulorhexis’, and ‘reading basics’.

The majority of trainees (82 %) reported that they had reflected on their performance following an OSATS. Overall the most positive aspects of the process identified were feedback, learning, and opportunity for reflective practice (Table 1). Comments included: ‘An opportunity for formal structured surgical feedback’, ‘ways to improve performance and resources to use e.g. the wet lab’, and ‘encourages reflective practice’.

**Table:** 

Year of training/number of years in ophthalmology:	
Deanery:	
How many times have you undertaken OSATS? 0 1 2 3 4+	
Who assessed you?	
Did you find it difficult to have the assessment done (e.g. case selection, time constraint)? If yes, please specify	
Do you feel OSATS is adaptable for every procedure including cataract and glaucoma surgery?	
Did you have any difficulties using the current format?	
Was sufficient time available to complete the procedure?	
Was sufficient time available to receive feedback?	
Was previous agreed action taken into consideration when filling in subsequent forms?	
Did you feel stressed during the assessment? If yes do you think it affected your performance in a negative way?	
Did any particular problems arise with the process (such as uncertainty on the correct way to perform an aspect of the procedure)? (Please specify)	
Have you been deemed unsatisfactory in a procedure overall or in any domain?	
Were any particular learning points identified (please specify)?	
Did it help you improve your skills?	
Did you reflect on your performance after completing this assessment?	
Was your performance in OSATS discussed in the ARCP?	
Overall, what were the good aspects of OSATS?	
Overall, what were the bad aspects of OSATS?	
How do you feel OSATS could be improved?	
Any other comments?	
Thank you for taking the time to fill out this questionnaire

**Table 1:** Main positive and negative themes of OSATS

Theme	Number of comments
Positive aspects	
Feedback	21
Learning points	23
Opportunity for reflective practice	23
Improvement of surgical skills	16
Negative aspects	
Time-consuming	11
Stressful	17
Assessor’s availability	2
Case-dependent	1

The most negative aspects of OSATS identified were time constraints, assessor’s availability and case selection (Table 1). Comments included: ‘difficult to find the time and the appropriate assessor’, ‘OSATS can place a strain on the relationship between consultants and trainees as it frequently necessitates that trainees pursue already busy consultants to complete the online assessment’, ‘places you under additional pressure when performing the procedure being assessed’, ‘no allocated time’, and feels more like ‘a tick-box exercise’.

## Discussion

The goals of assessment in ophthalmic surgery are to assess surgical skills, improve the surgical learning curve and identify factors that affect trainees’ surgical outcomes. Ideally assessment should identify underperforming doctors early in training who need extra support [4]. Trainees strongly value the opportunity to be observed by an expert and receive both positive and negative feedback. User satisfaction/acceptability for problem-based assessment/OSATS is highest for providing feedback and as an aid for learning as identified in a recent HTA [5].

It is therefore important that feedback on WpBAs is obtained from trainees to ensure problems with assessments are highlighted and solutions found.

The main problems encountered in the assessment were from time constraints and assessor availability. There were some issues with case selection, time to complete the case and stress related to the assessment, which have also been noted in previous studies [6–9]. There is no single way to solve the organizational issues and time factors involved in undertaking the assessments. Dialogue is encouraged between trainees and consultants to work out convenient local solutions. Reflecting this in job plans would allow more time and flexibility for trainers to supervise assessments and give feedback [8]. James et al. [10] found that in colorectal surgery commitment and planning helped make WpBAs feasible. Identification of appropriate cases pre-operatively, and allocating sufficient time immediately after the surgery for completion of the assessment were some suggestions which can be adapted for surgical assessments in ophthalmology. The most positive aspects of OSATS were improvement in surgical technique and feedback allowing for reflective practice. Trainees strongly value the opportunity to be observed by an expert and receive both positive and negative feedback. The majority of the senior trainees as opposed to junior trainees reported that insufficient time was allowed for feedback and almost half of them felt stressed during the process. This could be from higher expectations with seniority and is an area where demonstration of longitudinal evaluation of performance as discussed later will be useful.

More alarming was that 43 % of the trainees felt that OSATS did not help them to improve their surgical skills, and was perceived as a tick-box exercise. Some trainees also felt that their feedback was unhelpful. Furthermore, only a small minority of trainees felt that their WpBA results had had a significant bearing on the ARCP. A cultural change is needed for trainees to feel that WpBAs are not just a tick-box exercise, but a useful educational tool for learning. Training of clinical supervisors is required for good supervision and feedback. Discussion of key aspects from a trainee’s assessment (even if positive) should formulate part of the discussion during the ARCP.

Saleh et al. [11] have designed a specific assessment—objective structured assessment of cataract surgical skills—for assessment of anonymized surgical recording of cataract surgery, which is effective in differentiating between surgeons of varying experience as well.

Individualized OSATS for specific surgical procedures will help ensure better validity and video recording of assessments might help overcome observer bias [11]. Video-based virtual reality and dexterity systems should perhaps be used in conjunction with OSATS.

OSATS is currently most accepted as the ‘gold standard’ for objective assessment of surgical skills. However, there is no evidence on whether OSATS can distinguish between different levels of performance in the operating theatre. Surgeons are expected to show improvement with time but the present assessment system does not provide this information. This study found that previous assessments were not taken into consideration for subsequent assessments. A longitudinal evaluation of performance demonstrates progress (learning curve) over time [12]. The present assessments also need to incorporate some criteria to differentiate amongst trainees and identify means of encouraging and rewarding for excellence.

## Conclusion

A response rate of 83 % was achieved in this study. Lack of experience and questionnaire fatigue may have all impacted on the response rate. We do not feel that response bias is likely to be a significant problem in this study. It was reassuring that strong opinions both for and against the assessments were voiced, but many of the responses reflected moderate, balanced views. Small sample size is a limitation of the present study. The study, however, highlights important aspects of trainees’ perceptions of OSATS and identifies areas for improvement. To our knowledge this study is the first of its kind in the United Kingdom to seek trainees’ views on OSATS for assessing cataract surgery skills and the issues identified here will be relevant to other specialities as well. A larger survey would be beneficial.
